# Effect of Boron and Water-to-Cement Ratio on the Performances of Laboratory Prepared Belite-Ye’elimite-Ferrite (BYF) Cements

**DOI:** 10.3390/ma14174862

**Published:** 2021-08-26

**Authors:** Raquel Pérez-Bravo, Alejandro Morales-Cantero, Margherita Bruscolini, Miguel A. G. Aranda, Isabel Santacruz, Angeles G. De la Torre

**Affiliations:** 1 Departamento de Química Inorgánica, Cristalografía y Mineralogía, Facultad de Ciencias, Campus Teatinos, Universidad de Málaga, 29010 Málaga, Spain; pbr@uma.es (R.P.-B.); alejandrom@uma.es (A.M.-C.); g_aranda@uma.es (M.A.G.A.); isantacruz@uma.es (I.S.); 2 Dipartimento di Scienze Della Terra, Università Degli Studi di Milano, Via Botticelli 23, I20133 Milano, Italy; mbruscolini@rss-hydro.lu

**Keywords:** belite, calcium sulphoaluminate, rheology, superplasticisers, Rietveld method, degree of hydration, mechanical strengths

## Abstract

The effect of superplasticiser, borax and the water-to-cement ratio on BYF hydration and mechanical strengths has been studied. Two laboratory-scale BYF cements—st-BYF (with β-C_2_S and orthorhombic C_4_A_3_ $\bar{S}$) and borax-activated B-BYF (with α’_H_-C_2_S and pseudo-cubic C_4_A_3_ $\bar{S}$)—have been used, and both show similar particle size distribution. The addition of superplasticiser and externally added borax to BYF pastes has been optimised through rheological measurements. Optimised superplasticiser contents (0.3, 0.4 and 0.1 wt % for st-BYF, B-BYF and st-BYF with externally added 0.25 wt % B_2_O_3_, respectively) result in low viscosities yielding homogeneous mortars. The calorimetric study revealed that st-BYF is more reactive than B-BYF, as the values of heat released are 300–370 J/g and 190–210 J/g, respectively, after 7 days of hydration; this fact is independent of the water-to-cement ratio. These findings agree with the higher degree of hydration at 28 days of β-C_2_S in st-BYF (from 45 to 60%) than α’_H_-C_2_S in B-BYF (~20 to 30%). The phase assemblage evolution has been determined by LXRPD coupled with the Rietveld method and MAS-NMR. The formation of stratlingite is favoured by increasing the w/c ratio in both systems. Finally, the optimisation of fresh BYF pastes jointly with the reduction of water-to-cement ratio to 0.40 have allowed the achieving of mortars with compressive strengths over 40 MPa at 7 days in all systems. Moreover, the st-BYF mortar, where borax was externally added, achieved more than 70 MPa after 28 days. The main conclusion of this work does not support Lafarge’s approach of adding boron/borax to the raw meal of BYF cements. This procedure stabilises the alpha belite polymorph, but its reactivity, in these systems, is lower and the associated mechanical strengths poorer.

## 1. Introduction

The need of reducing the environmental impact of the cement industry is a hot topic [1,2]. Belite-ye’elimite-ferrite (BYF) cements are considered as green materials due to the low energy demand and low CO_2_ emissions of their production process [3,4] compared to Portland cement (PC). The production of these materials is feasible [5] and the use of alternative raw materials is a step forward in sustainability [6,7,8]. However, belite-rich cements are still a challenge [9] due to their lower reactivity rate with water and the consequent lower early mechanical strengths development. Additionally, these cements have a lower degree of grindability, requiring higher amounts of energy for a targeted fineness [10]. One of the approaches to enhance the reactivity of BYF has been the stabilisation of high temperature polymorphs of belite, i.e., α-forms, at room temperature by the inclusions of minor elements such as sodium, potassium or sulphur [11,12] or boron [13,14,15]. There is another activation route consisting of reducing the particle size by milling or increasing the defects/strains in the particles by rapid cooling rates during clinkering [16,17].

The hydration mechanisms of these types of materials have been studied in the past years. The kinetic of hydration of the ye’elimite phase is very fast and almost independent of the amount of anhydrite or water-to-cement ratio [18] but it can be influenced by the presence of other aluminates [19]. In addition, the sulphate content and type affect the kinetic of silicates in general [20,21], and belite in particular [18,22], by retarding their hydration with the increase in sulphate dissolution. The decrease of water-to-cement (w/c) ratio is a positive strategy to enhance mechanical strengths [23,24] but its effect on hydration kinetics is still not fully understood, especially the effect on phase assemblage and microstructural development [25]. Moreover, the relationship among the complex phase assemblage, the microstructure and the mechanical properties is still a challenge in common systems [26,27], and even more in BYF.

Furthermore, mortars with optimised performances can be prepared through the control of the physical–chemical properties involved in fresh cementitious systems. The required homogeneity (and workability) of fresh cementitious systems can be achieved through the addition of admixtures such as superplasticisers (SPs), which is a common practice in the building industry. SPs are usually adsorbed onto the surface of the cement particles, helping the dispersion of the particles through electrostatic and/or steric repulsions. The effectiveness of the SP depends on the properties of the materials (cement composition, specific surface area, size, shape of particles and type of SP), the amount of added SP, and the preparation of the paste (order of addition, time passed since the end of mixing, temperature and so on). SPs based on polycarboxylate show important advantages in the dispersion of cement particles over others, such as lignosulfonate, melamine or naphthalene-based SPs [28]. Thus, SPs allow the preparation of mortars with low w/c ratios with competitive or even enhanced performances since they help the dispersion of the cement particles and reduce viscosity, can also modify the setting time, and will affect the strength development (and durability) of final mortars and concretes [29]. Hence, the optimisation of the amount (and type) of this chemical admixture is key to achieve the objective of improving the performances of the final mortars [30].

In this article, two BYF materials, one with β-C_2_S and another one with α’_H_-C_2_S have been characterised with the objective to unravel the influence of admixtures, i.e., superplasticiser and borax, and the water-to-cement ratio on the hydration mechanisms and mechanical strengths. Rheological studies have been performed to understand the fresh state of these materials. Calorimetric measurements, X-ray diffraction (with Rietveld analysis) and MAS-NMR studies have been performed to understand the role of admixtures and water on the heat evolved and phase assemblage. Moreover, mechanical strengths of some selected mortars have been tested and correlated with the phase development.

Cement nomenclature will be used hereafter: C = CaO, S = SiO_2_, F = Fe_2_O_3_, A = Al_2_O_3_, $\bar{S}$ = SO_3_ and H = H_2_O.

## 2. Materials and Methods

### 2.1. Cement Preparation

Two kilograms of two BYF clinkers, standard and activated, were prepared as reported elsewhere [31]. The former, without activation, was called st-BYF, and the latter, called B-BYF, was prepared by adding borax in the clinkering step. The elemental compositions of the clinkers are given in Table 1.

Both clinkers were grinded with 10 wt % of anhydrite to prepare the cements [22]. The anhydrite was prepared by heating commercial basanite (BELITH S.P.R.L., Mouscron, Belgium) at 700 °C for 60 min. Air permeability values of the final cements were 5650 and 5260 cm^2^/g for st-BYF and B-BYF, respectively. Figure S1, provided as supplementary information, shows the particle size distribution measured by laser diffraction (Mastersizer 3000, Malvern, UK). The equipment was provided with a wet module with ethanol and is located in Servicios Centrales de Apoyo a la Investigación (SCAI) at Universidad de Malaga (UMA) (Malaga, Spain). D_v,10_, D_v,50_ and D_v,90_ values were 2.0, 7.4 and 27.5 µm, and 2.7, 8.3 and 30.4 µm, for st-BYF and B-BYF, respectively.

### 2.2. Paste Preparation

Cement pastes were prepared with deionised water (w/c = 0.40 and 0.50) by mechanical stirring at a fixed speed of 800 rpm during 90 s twice with a 30 s resting time in between.

To assure homogeneity of the pastes, a commercial polycarboxylate-based superplasticiser (SP), with a 25 wt % of active matter (Floadis 1623 marketed by Adex Polymer S.L., Madrid, Spain) was added when appropriate. The amount of the added superplasticiser was always shown as active matter of the SP referred to cement content, and the extra water added was considered for w/c calculations. SP was always added to the water prior to the cement addition. Borax, as admixture, was also used with selected st-BYF pastes with w/c = 0.40, by adding it to the water (after the addition of SP) until total dissolution (named hereafter as st-BYF-B_2_O_3_).

The pastes were poured into polytetrafluoroethylene (PTFE) cylindrical moulds that were hermetically closed and rotated (16 rpm) during the first 24 h at 20 ± 1 °C. Then, the cylinders were demoulded and placed into a bath of water at 20 ± 1 °C to be used at the selected hydration ages (7, 28 and 120 days). The hydration of the samples was arrested before characterisation by manually grinding and washing powders twice with isopropanol and once with diethyl ether, and finally they were dried at 40 °C for 24 h in a stove.

### 2.3. Laboratory X-ray Powder Diffraction (LXRPD) and Data Analysis

Anhydrous cements and arrested-hydration pastes were analysed by LXRPD collected on an X’Pert MPD PRO PANalytical diffractometer with copper radiation with a Johansson Ge(111) primary monochromator that yields monochromatic CuKα_1_ (λ = 1.5406 Å). The diffractometer is located at SCAI at UMA (Malaga, Spain). Data were collected on reflection geometry from 5° to 70° (2θ) with a measuring time of 2.5 h by using a rotating sample-holder (16 rpm).

The patterns were analysed by the Rietveld method using the GSAS software package [32], using a pseudo-Voigt peak shape function with the asymmetry correction included [33,34] to obtain RQPA (Rietveld quantitative phase analysis). The refined overall parameters were: background coefficients, zero-shift error, phase scale factors, unit cell parameters, peak shape parameters and preferred orientation coefficient, if needed. The structure descriptions of crystalline anhydrous and hydrated phases used are given elsewhere [35,36]. The determination of amorphous and crystalline non-quantified (ACn) was performed by the internal standard methodology [37], mixing the samples with ~20 wt % of crystalline quartz.

### 2.4. Thermogravimetric Analysis

The free water (i.e., non-chemically combined water) content of all pastes at all ages was calculated by analysing the thermogravimetric traces of all arrested-hydration pastes. An SDT-Q600 analyser (TA Instruments, New Castle, DE, USA) was used to perform these analyses. Some milligrams of arrested-hydration pastes were placed in open platinum crucibles under air flow and heated from room temperature (RT) to 1000 °C (at 10 °C/min). The free water was calculated as detailed in [38] by considering the weighed loss from RT to 600 °C as chemically bounded water and the one from 600 to 1000 °C as CO_2_.

### 2.5. Rheological Behaviour of Cement Pastes

st-BYF, B-BYF and st-BYF-B_2_O_3_ (the latter with 0.25 wt % B_2_O_3_, referred to dry cement) pastes with different SP contents (from 0.0 to 0.5 wt %) at w/c = 0.40 were rheologically characterised.

The rheological characterisation was performed in a viscometer (Model VT550, Thermo Haake, Karlsruhe, Germany) with a serrated coaxial cylinder sensor, SV2P, which was provided with a lid to reduce evaporation. Two different measurements were performed: (i) flow curves, at controlled rate, from 2 to 350 s^−1^, for a total of 12 ramps (up-curve), with ramp times of 6 s. A further decrease from 350 to 2 s^−1^ shear rate was performed by following the same ramp times (down-curve). Pastes were pre-sheared at 350 s^−1^ for 30 s and held at 0 s^−1^ for 5 s before the measurement. Data were acquired after 8 min since the cement powder was added to water; (ii) viscosity vs. time measurements at the fixed shear rate of 5 s^−1^.

### 2.6. Magic Angle Spinning Nuclear Magnetic Resonance (MAS-NMR) Study

^29^Si MAS-NMR spectra of all samples were recorded at room temperature on a Bruker AVIII HD 600 NMR spectrometer(Karlsruhe, Germany) (field strength of 14.1 T) at 119.8 MHz with a 2.5 mm triple-resonance DVT probe using zirconia rotors at 15 kHz spinning rate, located in SCAI at UMA (Spain). The experiments were performed with ^1^H decoupling by applying single-pulse excitation with a π/2 pulse of 5 us, 30 s relaxation delay and 10,800 scans. The chemical shift was referenced to an external solution of tetramethylsilane (TMS).

^27^Al MAS-NMR spectra were recorded in the same spectrometer at 156.4 MHz and the rotors operated at 20 kHz. The experiments were performed with and without ^1^H decoupling (cw sequence) by applying a single pulse (π/12), an excitation pulse of 1 μs and 5.0 s relaxation delay and 200 scans. The chemical shift was referenced to an external solution of 1 M of Al(NO_3_)_3_.

### 2.7. Isothermal Calorimetry

The isothermal calorimetric measurements were performed in an eight channel Thermal Activity Monitor, of TA Instruments (New Castle, DE, USA), using glass ampoules of 20 mL. Firstly, ~8 g of anhydrous cements was manually mixed with the corresponding amount of water (and superplasticiser) for 1 min followed by an extra minute of stirring with a vortex mixer. Then, the paste was placed in the ampoule (~6 g). Water was used as reference [39]. The heat flow data were collected for up to 7 days at 20 °C. The first 45 min after inserting the ampoules in the equipment were not recorded as this time was needed for its stabilisation.

### 2.8. Mortar Preparation

Mortars were prepared according to UNE-EN196-1 at the cement/sand mass ratio of 1/3 and w/c ratios of 0.40 for all the samples, and additionally, 0.50 for B-BYF. Mortars were prepared with selected amounts of superplasticiser (0.3, 0.4 and 0.1 wt % referred to cement, for st-BYF, B-BYF and st-BYF-B_2_O_3_, respectively). The compression strengths were measured on mortar cubes (3 × 3 × 3 cm^3^), cast and cured at 20 ± 1 °C and 99% relative humidity (RH) for 24 h. The cubes were demoulded and kept in a water bath (20 ± 1 °C) for 7, 28 and 120 days of hydration. Three cubic mortars were tested at each hydration age and the value given is the average value with the corresponding standard deviation (UNE-EN196-1). The values given in this study are the result of applying a correction factor (1.78) to compare them with the values obtained with standard prisms (4 × 4 × 16 cm^3^). Mortars prepared with Portland cement (PC type I 42.5R from Financiera y Minera S.A (Malaga, Spain) which belongs to Heidelberg Cement Group) are also shown for the sake of comparison.

## 3. Results and Discussions

### 3.1. Rheological Study

**Figure 1:**
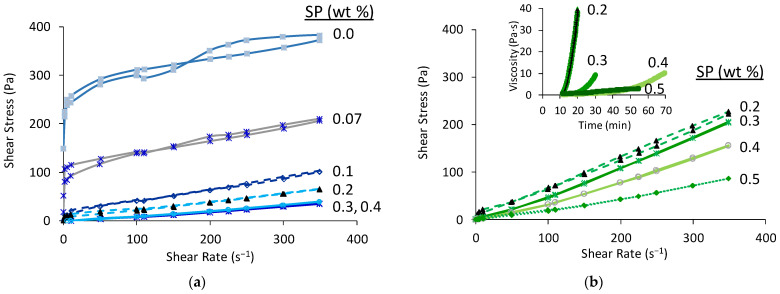
Flow curves of (**a**) st-BYF and (**b**) B-BYF pastes prepared at w/c = 0.40 and different superplasticiser (SP) contents. Inset of (**b**) shows the evolution of the viscosity with time of B-BYF pastes (w/c = 0.40) and different amounts of SP at a shear rate of 5 s^−1^. Numbers close to each curve stand for the amount of SP (wt %) of active matter referred to cement content.

The amount of admixtures, SP and externally added borax was optimised in st-BYF and B-BYF pastes prepared with w/c of 0.40. Figure 1a,b shows the flow curves of st-BYF and B-BYF pastes, respectively, prepared with different SP contents. As expected, the shear stress, and consequently, the viscosity of both families decreases by increasing the SP content up to a certain point, which corresponds to the “saturation point”. In the case of st-BYF pastes, minimum values of viscosity, and hence better homogenisation, are achieved with the addition of 0.3 wt % SP; when the SP content was increased to 0.4 wt %, similar viscosity values were achieved, so the value of 0.3 wt % was selected as the optimum one.

B-BYF pastes show higher viscosity values than the corresponding st-BYF pastes for the same SP contents and need higher contents of SP to improve its workability. As the particle size is similar in both cases (Figure S1), and the same procedure was followed in the preparation of both families, the difference is attributed to the nature and composition of the powders. Here, the paste with 0.5 wt % SP shows lower viscosity than the paste with 0.4 wt %. No higher amounts of SP were studied because polycarboxylate-based superplasticisers act also as retarders [5,38,40], and the viscosity of the paste was low enough to prepare homogeneous specimens. To further investigate this effect, the evolution of the viscosity with time was studied at the shear rate of 5 s^−^^1^ (inset Figure 1b). This shear rate was selected as it is high enough to obtain accurate data, and low enough for not altering considerably the structure of the pastes [38,40]. In all cases, the viscosity increases with time but that rising is delayed by the addition of SP, as mentioned before. As the addition of 0.5 wt % delays considerably the evolution of viscosity, the amount of 0.4 wt % of SP was selected for further studies, and no extra additions were studied.

**Figure 2:**
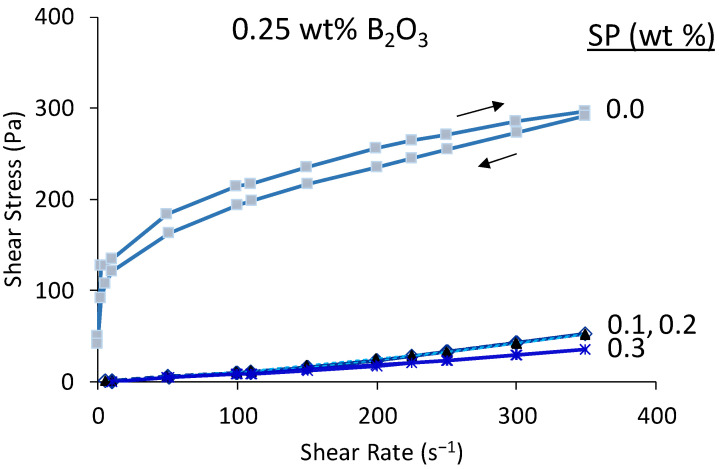
Flow curves of st-BYF with 0.25 wt % of B_2_O_3_ externally added as borax with w/c = 0.40 and different superplasticiser (SP) contents.

The effect of B_2_O_3_, added as borax to st-BYF, was studied in order to unravel if the external addition is a feasible strategy of activation. The first attempt was to add the same amount of boron as B-BYF contains (i.e., 2.0 wt % of B_2_O_3_) but, as expected [15], the pastes did not set even after 1 day of hydration. Consequently, the addition of 0.5 wt % of B_2_O_3_ in the paste was tested, with the optimised amount of SP, 0.3 wt % SP; however, pastes were still fluid 24 h later, even when lower amounts of SP were added. Thus, the amount of B_2_O_3_ had to be reduced to 0.25 wt %, and the amount of SP was optimised for this system. Figure 2 shows the flow curves of the st-BYF-0.25B_2_O_3_ paste with different SP contents (from 0.0 to 0.3 wt %). The addition of a small amount of SP, 0.1 wt %, reduces considerably the viscosity, and higher additions, 0.2 and 0.3 wt %, do not have an important improvement to the viscosity. Thus, the amount of 0.1 wt % SP was selected for further studies.

**Figure 3:**
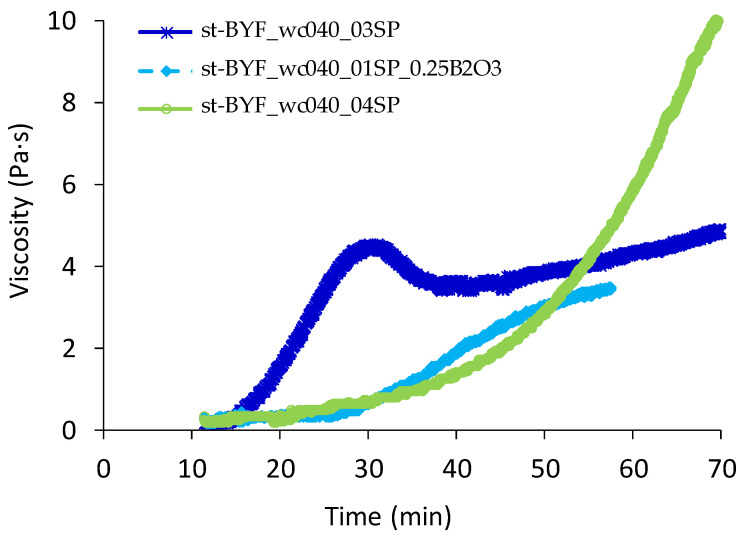
Evolution of the viscosity with time of st-BYF_wc040_03SP, st-BYF_wc040_01SP_0.25B_2_O_3_ and B-BYF_wc040_04SP pastes at a shear rate of 5 s^−^^1^.

It is important to prepare homogeneous pastes and mortars with low initial viscosity, but also with a controlled rising in viscosity to have enough open time to set in place homogeneous pastes and mortars that set at an economically feasible range of time. Because of that, the evolution of the viscosity with time of the three selected families (st-BYF_wc040_03SP, st-BYF_wc040_01SP_0.25B_2_O_3_ and B-BYF_wc040_04SP) is shown in Figure 3, where the viscosity of all pastes rises with time. Although the initial viscosity of the optimised st-BYF paste (0.3 wt % SP) is lower than the selected B-BYF paste (0.4 wt % SP) (Figure 1), the viscosity of the former increases more quickly during the first 55 min (Figure 3).

In addition, the st-BYF paste suffers a decreasing in viscosity, from 28 to 32 min of hydration, which is attributed to a stable zone where the aggregates are being broken by shear [41]; after that, viscosity increases again due to the evolution of the hydration. The rise in viscosity of st-BYF-0.25B_2_O_3_ paste is slower than the corresponding one for st-BYF paste, even with a lower amount of SP, very likely due to the retarder effect of borax [15].

### 3.2. Calorimetric Study

**Figure 4:**
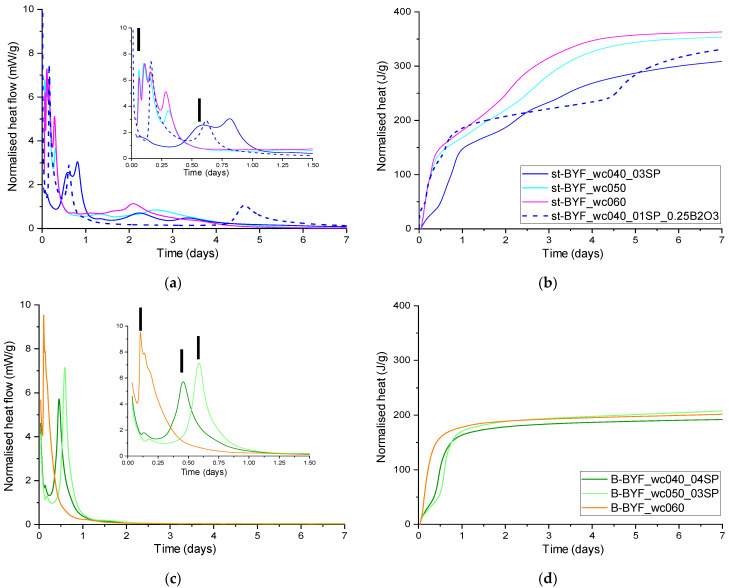
Calorimetric curves for (**a**) and (**b**) st-BYF and (**c**) and (**d**) B-BYF, with different w/c ratios. Vertical bars in (**a**) and (**c**) indicate the heat evolved due to the precipitation of AFt.

Figure 4 shows the calorimetric curves of all the studied cements with different w/c ratios. Pastes were prepared with the optimised amount of superplasticiser determined in the previous section. In both families, st-BYF and B-BYF, there are intense signals in the first 24 h, corresponding to the dissolution of ye’elimite and anhydrite and the precipitation mainly of ettringite [25]. These results have to be discussed according to different variables: (i) effect of mineralogy of st-BYF and B-BYF, (ii) effect of w/c ratio in each family and (iii) effect of admixtures: superplasticiser and borax.

(i). Mineralogy effect: The most significant effect, which is mainly due to the different mineralogy, is the total heat released after 7 days, being well over 300 J/g in all st-BYF pastes and close to 200 J/g in all the B-BYF pastes. This means that st-BYF is more reactive than B-BYF at early ages. To detail this discussion, both pastes with w/c of 0.60 pastes were compared, because with this amount of water, superplasticiser was not required. The heat released by both st-BYF-wc060 and B-BYF-wc060 is ~150 J/g after 12 h of hydration (Figure 4b,d), indicating that the dissolution of ye’elimite and anhydrite in both families have taken place at the very same pace. However, st-BYF presented an increase on heat released after 24 h of hydration. This effect is usually related to the formation of AFm [25,42] but in these systems, it is mainly due to the precipitation of calcium aluminosilicate hydrates such as stratlingite or iron-siliceous hydrogarnet [43,44] from the dissolution of β-C_2_S [45], as will be discussed later.
(ii). Effect of w/c: On the one hand, st-BYF-wc050 and st-BYF-wc060 present four signals before 12 h of hydration, almost coincidentally (Figure 4a). For Portland cements, it has been described that the increase of w/c ratio slightly delays the very early hydration [46]. In these systems, after 3 h of hydration, the heat released has been 43.9 and 39.7 J/g for w/c of 0.50 and 0.60, respectively, confirming the slight retardation also for this BYF system. This could be due to the higher alkaline contents in the pore solution for lower w/c ratios. Moreover, the values of the total heat released after 7 days are 353 and 363 J/g, respectively; this is also in agreement with the slightly higher hydration degree at later ages by increasing the w/c ratio. However, compared to st-BYF_wc040_03SP, with w/c 0.40, the total heat released after 7 days is ~14% smaller, indicating the lower degree of hydration. This may be due to the lower w/c ratio [46]. On the other hand, B-BYF with w/c of 0.40, 0.50 and 0.60 released almost the same heat after 7 days of hydration; on average 200(6) J/g, indicating that the w/c ratio has little impact in this system at these intermediate ages.
(iii). Effect of admixtures: The addition of superplasticiser retards the hydration in all cases as expected [47,48] (Figure 4). The first signal of all calorimetric curves is assigned to the precipitation of AFt, which has been delayed from 1.4 h in st-BYF pastes without any SP to ~14 h for the sample st-BYF_wc040_03SP (vertical bars in Figure 4a). The addition of SP to B-BYF pastes has also delayed the hydration (Figure 4b).

St-BYF_wc040 was also hydrated with borax as admixture to test its effect on hydration. This sample also needs SPs due to the low w/c ratio of 0.40. However, as detailed in the previous section, the amount of SP was reduced from 0.3 wt % (that was added to st-BYF with w/c of 0.40 without borax) to 0.1 wt % SP for the paste with 0.25 wt % B_2_O_3_. As expected, the addition of boron (with SP) has retarded the hydration [15] by separating the dissolution/precipitation phenomena into two signals, one centred at ~3.8 h and the other one at ~14.6 h. The former may be mainly related to the precipitation of AFt and the latter to the dissolution and precipitation of silicon-bearing phases; this deserves in situ research that will be published elsewhere. This future research will help to understand the specific role of admixtures by disentangling the effect on each individual hydration phenomena.

### 3.3. Mineralogical Evolution with Time: LXRPD and MAS-NMR Study

Figures S2–S8 give the raw LXRPD patterns of all pastes at all studied hydration ages. Figures S9–S11 give the Rietveld plots of selected pastes as representative examples. Table 2, Table 3, Table 4, Table 5, Table 6, Table 7 and Table 8 give RQPA results of all pastes for all the studied ages, expressed in weight per 100 g of dry cement. These tables also include the ACn content, and the free water calculated from the thermal analysis as detailed in Section 2.

In all samples, ye’elimite has completely reacted after 7 days. However, none of the pastes have reached the theoretical amount of ettringite according to reaction (1) [49,50], being 30.5 and 35.0 g/100 g of dry cement for st-BYF and B-BYF, respectively, independently of the amount of water (with the limiting phase being ye’elimite). In fact, all the pastes with w/c 0.50 and 0.60 contain a certain amount of free water, i.e., non-reacted water, meaning that the availability of water was not the reason of not reaching the theoretical amount of crystalline ettringite.

(1) $$C_{4}A_{3}\bar{S}+2C\bar{S}+\left(38+2x\right)H\;\to \;C_{6}A{\bar{S}}_{3}H_{32}+2AH_{3}\cdot xH,$$

This fact means that the amorphous/nanocrystalline fraction (ACn content) contains not only the aluminium hydroxide gel but also nano-crystalline sulphate-bearing phases. The average amount of crystalline AFt after 28 days is 13.1(3.9) and 22.1(0.5) g/100 g of dry cement for st-BYF and B-BYF families, respectively. These values mean only ~40% and ~63% of the theoretical amount of AFt that could precipitate are present as crystalline components. After 120 days, the mean crystalline ettringite has increased up to 17.4(0.9) and 31.4(1.5) g/100 g of dry cement for st-BYF and B-BYF families, respectively, being ~57% and ~90% of the theoretical amount of AFt that could precipitate since ye’elimite was dissolved completely.

**Figure 5:**
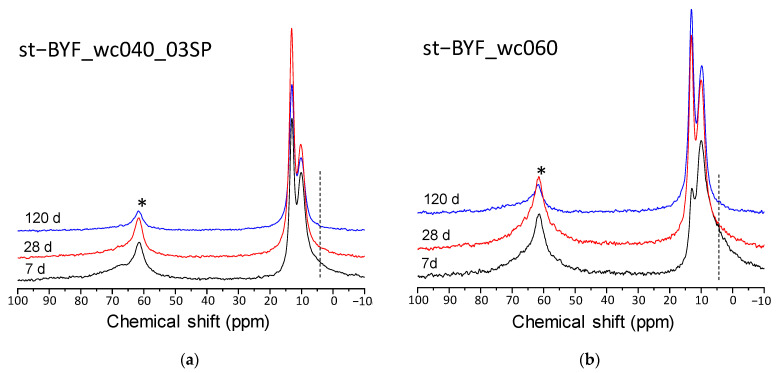
^27^Al MAS-NMR spectra for (**a**) st-BYF_wc040_03SP and (**b**) st-BYF_wc060 as a function of time. Asterisk indicates the interlayer ^IV^Al resonance due to stratlingite. Vertical dashed lines mark the resonance due to ^VI^Al in AH_3_-gel.

Figure 5 displays ^27^Al MAS-NMR spectra for st-BYF pastes with w/c 0.40 and 0.60 as a function of time as illustrative examples of the effect of w/c ratio on phase assemblage. After 7 days of hydration, the characteristic resonance bands of octahedrally coordinated Al at ~13 and ~5 ppm of AFt and AH_3_-gel or “third aluminate hydrate” [50], respectively, are present in all the spectra. A recent study of Portland cement with calcined clays has determined that the latter signal is mainly due to bridging Al(VI) species which behave as network formers in C-A-S-H gel [51]. However, in these aluminate rich systems the presence of ill-crystallised AH_3_ has been confirmed [52]. The signal due to this AH_3_-gel in the systems with higher w/c ratio are more intense and broader (see dashed lines in Figure 5). It has been demonstrated [25] that the kinetic of hydration of ye’elimite is not modified by the variation of the w/c ratio, and here what we are observing is that the increase of the w/c ratio has affected the precipitation of crystalline phases, with the amorphous component being higher at 7 days (Table 2; Table 5). Interestingly, the ratio of stratlingite/katoite increases by increasing the w/c ratio, meaning that at a low w/c ratio the formation of stratlingite is impaired, as previously observed [25,53], but the crystallisation of iron-bearing katoite is favoured (Table 2, Table 3, Table 4, Table 5, Table 6, Table 7 and Table 8).

**Figure 6:**
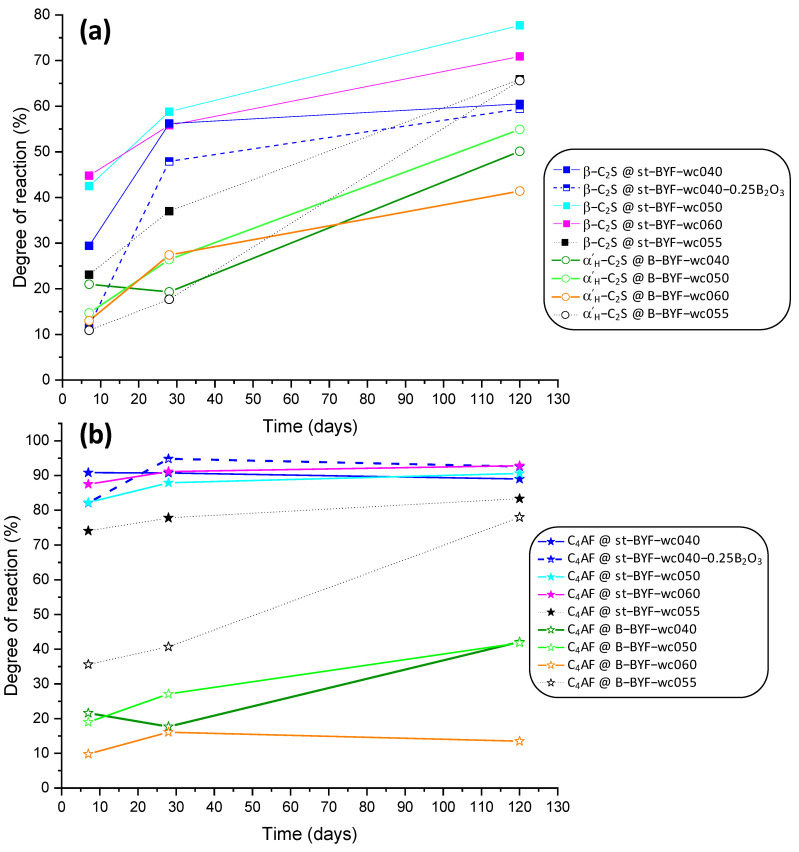
Degree of reaction of (**a**) β-C_2_S and α’_H_-C_2_S and (**b**) C_4_AF in all BYF pastes. Data calculated from [22] for w/c of 0.55 are also included.

Moreover, the degree of reaction of both β-C_2_S and α’_H_-C_2_S, in all pastes, has been calculated from data given in Table 2, Table 3, Table 4, Table 5, Table 6, Table 7 and Table 8, and is shown in Figure 6a. The latter is always lower than the former for all pastes, in agreement with calorimetric results. Moreover, the dissolution rate of C_4_AF is directly correlated with that of belite; consequently, the degree of hydration of the ferrite phase in st-BYF is also higher at any w/c ratio (see Figure 6b). The data obtained in a previous study [22] for pastes prepared with w/c of 0.55 are also included for the sake of comparison and agree with the results reported here. The higher hydration rate of β-C_2_S than α’_H_-C_2_S may be due to the higher volume of the former due to the presence of sulphur [54,55], i.e., 347.4(1) Å^3^ compared to 345.8 Å^3^, which was stabilised with 0.5 wt % of Cr_2_O_3_ [56]. Consequently, the approach of adding boron or borax reported by Lafarge [57,58] to stabilise α’_H_-C_2_S, which is a-priori highly reactive, is not supported by this study since these polymorphs present a lower hydration degree at any w/c ratio or time.

The reactivity of β-C_2_S and ferrite can be described by reactions (2) and (3), being the formation of stratlingite and iron-containing katoite or siliceous hydrogarnet [43], respectively.

C_2_S + 2AH_3_ + 5H → C_2_ASH_8_, (2)

C_2_S + ½C_4_AF + 5H → C_3_S(A,F)_2_H_4_ + CH, (3)

**Figure 7:**
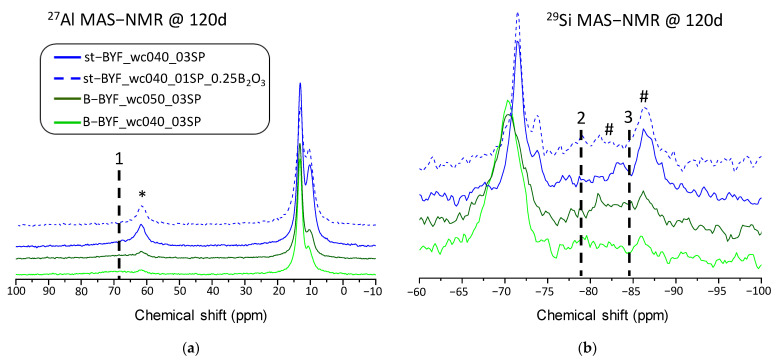
(**a**) ^27^Al MAS-NMR and (**b**) ^29^Si MAS-NMR spectra for selected pastes after 120 days of hydration. Asterisk indicates the interlayer ^IV^Al resonance due to stratlingite. # stands for Q^3^(2Al) and Q^2^(1Al) resonances of stratlingite. ^IV^Al resonance in C-A-S-H gel is labelled as 1 in (**a**). Q^1^ and Q^2^ resonances due to C-S-H gel are labelled as 2 and 3 in (**b**).

All st-BYF pastes, at any w/c ratio and any hydration time, present higher amounts of both stratlingite and katoite due to the higher degree of reaction of belite and ferrite. The presence of these phases has been confirmed also by NMR since stratlingite displays a resonance located at ~60 ppm (asterisks in Figure 5; Figure 7a), which correspond to the interlayer IV-coordinate-Al [59,60]; it enables us to clearly identify it by ^27^Al MAS-NMR. The resonance centred at ~9.5 ppm is due to VI-coordinated-Al, which is present in stratlingite, katoite and AFm phases. This signal is even larger than the AFt one after 7 days in st-BYF_wc060 (Figure 5b). Figure 7b also shows the ^29^Si MAS-NMR spectra of selected pastes; concretely the ones that were tested by mechanical strengths, after 120 days of hydration. The most remarkable difference among these spectra is the signal centred at approximately −86 ppm and approximately −82 ppm, labelled as # in Figure 7b, corresponding to Q^3^(2Al) and Q^2^(1Al) of stratlingite [61], respectively, present mainly in st-BYF pastes. The cohesive effect of stratlingite (and katoite) is still unknown, and further research is needed. Moreover, after 120 days of hydration, the signals due to C-S-H gel, at approximately −77 ppm and approximately −85 ppm for Q^1^ and Q^2^, respectively, are present in all the systems, labelled as 2 and 3 in Figure 7b. However, the broad resonance due to the IV-coordinated aluminium present in the C-A-S-H gel [59,62], which is labelled as 1 in Figure 7a, indicates that, only in the family of B-BYF, this gel contains a small amount of aluminium. This is understandable, since reactions (2) and (3) in st-BYF systems have taken place at a larger pace than in B-BYF systems, consuming aluminium. According to these data, it cannot be stated that the formation of C-S-H or C-A-S-H gel is favoured in st-BYF or in B-BYF, since ^29^Si MAS-NMR spectra are very similar.

**Figure 8:**
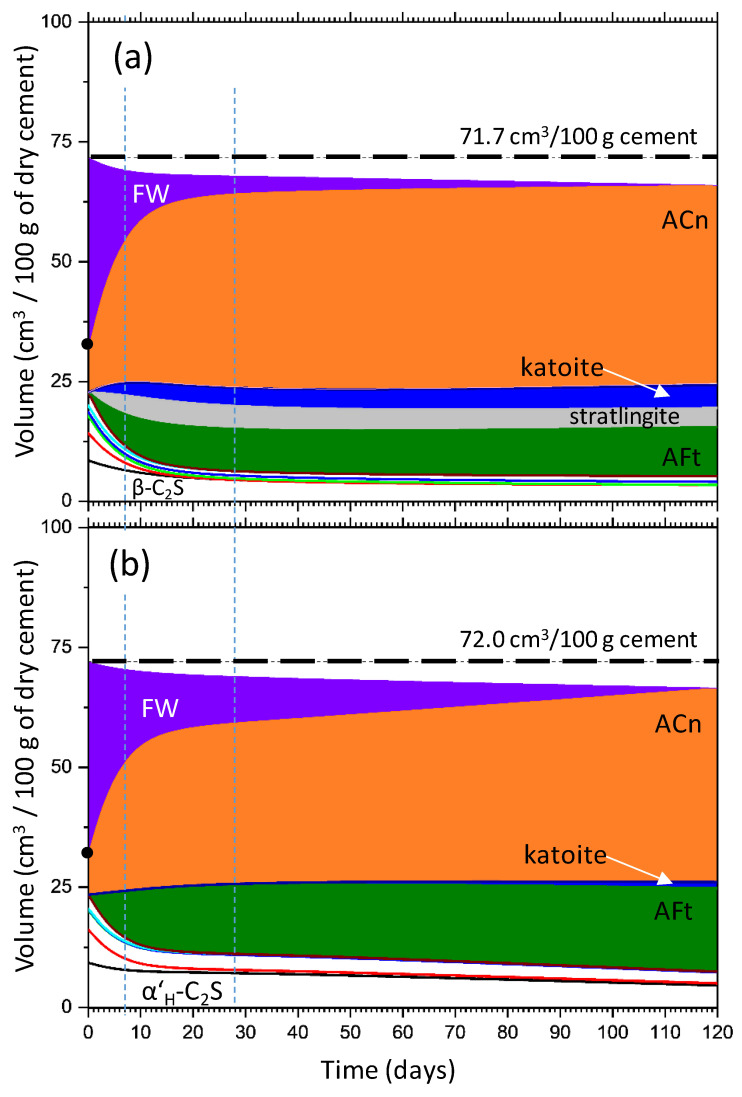
Volume evolution with time for (**a**) st-BYF-wc040_03SP and (**b**) B-BYF-wc040_04SP. Dashed vertical lines indicate 7 and 28 days of hydration.

The volume changes with hydration time may justify the mechanical performances. Thus, Figure 8 shows the volume evolution of two selected pastes, st-BYF-wc040_03SP and B-BYF-wc040_04SP as representative examples. The density values used to make these calculations are included in Table 2; Table 6. The volume increase due to the formation of crystalline ettringite, from 7 to 120 days, in both systems are 12.5 and 35.3%, for st-BYF-wc040_03SP and B-BYF-wc040_04SP, respectively. These increments of crystalline ettringite may be described as a crystallisation or crystal growth process from amorphous/nanocrystalline ettringite-like fraction to crystalline AFt. This crystal growth has happened after setting and consequently, it may cause internal stress that up to a certain level may enhance the mechanical properties [63], but if a threshold level is crossed the mechanical properties may be negatively affected [64]; see below in the mechanical strengths development. At this stage, more research is needed to develop quantitative techniques [65,66] to unravel the evolution of the microstructure of hydrating materials at the different length scales, from nano- to millimetres.

### 3.4. Mechanical Strengths

**Figure 9:**
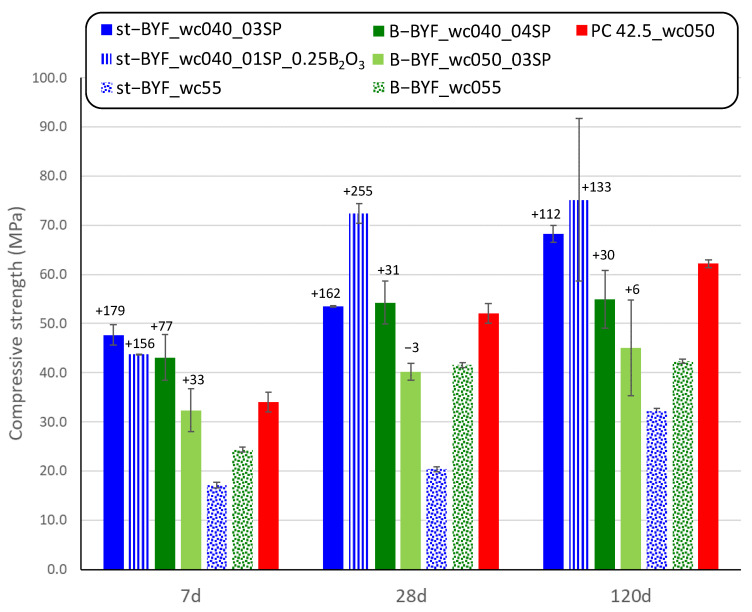
Compressive strengths of selected st-BYF and B-BYF mortars. Data from [22] and a type I 42.5R PC prepared as UNE-EN196-1 are also given for the sake of comparison. Percentage of mechanical strengths with respect to those with w/c of 0.55 from [22].

Figure 9 shows the compressive strength values of selected mortars of st-BYF and B-BYF. The reader has to bear in mind that these are laboratory-prepared cements, and consequently, not all the formulations could be tested by mechanical strengths. The water-to-cement ratio of 0.40 was selected for all cements to be compared with those published by our research group with w/c 0.55 [22]; in addition, B-BYF with w/c 0.50 was also tested. The first important result is that the reduction of w/c ratio from 0.55 to 0.40 causes an increase in the compressive strengths in both systems, st-BYF and B-BYF. The increase (in percentage) referred to the corresponding mortars prepared at w/c = 0.55 [22] are also included in Figure 9.

The higher degree of hydration of st-BYF phases and the optimisation of fresh pastes through rheological studies have caused the development of higher mechanical strengths after 7 days for all the tested samples, being competitive with a PC type I 42.5R. The workability at the fresh state of the mortars has a very important impact on the mechanical strengths, in agreement with previous studies [38]. These results also indicate that the mineralogical evolution with time, jointly with the degree of hydration of main phases, are useful to predict the mechanical strengths. In this study, B-BYF based mortars, with α’_H_-C_2_S, developed lower mechanical strengths than the optimised st-BYF mortars. These results could be predicted by the lower degree of hydration of the main phases.

## 4. Conclusions

Two laboratory-prepared BYF clinkers, standard (st-BYF) and activated with borax (B-BYF), were mixed with 10 wt % anhydrite to prepare BYF cements. As both clinkers show similar particle size distribution, the main difference between them is related to the belite and ye’elimite polymorphisms, where β-C_2_S and orthorhombic C_4_A_3_$\bar{S}$ are present in the former, and α’_H_-C_2_S and pseudo-cubic C_4_A_3_$\bar{S}$ are the main phases in the activated BYF. The external addition of borax to st-BYF pastes has also been studied. Homogeneous pastes and mortars were prepared after the optimisation of the superplasticiser content (0.3, 0.4 and 0.1 wt % for st-BYF, B-BYF and st-BYF with 0.25 wt % B_2_O_3_, respectively) through rheological measurements. As the external addition of borax resulted in the delay of hydration, the maximum amount of B_2_O_3_ that could be added was 0.25 wt % (pastes with 0.5 wt % B_2_O_3_ were still fluid even after 24 h).

The calorimetric study revealed that st-BYF is more reactive than B-BYF after 7 days of hydration. The hydration degree of the st-BYF paste slightly increased at later ages by increasing the w/c ratio, although B-BYF pastes prepared at different w/c ratios released almost coincident heat after 7 days of hydration.

The quantitative phase assemblage with time determined by LXRPD (and the Rietveld method) has enabled us to determine the higher degree of hydration at 28 days of β-C_2_S in st-BYF, from 45 to 60%, than α’_H_-C_2_S in B-BYF (~20 to 30%). Moreover, ye’elimite was completely reacted after 7 days in all samples, although none of the pastes reached the theoretical amount of ettringite; thus, the amorphous/nanocrystalline fraction (ACn content) contains not only the aluminium hydroxide gel but also nano-crystalline sulphate-bearing phases. The increase of volume due to the crystallisation of AFt, from 7 to 120 days, is ~13% in st-BYF and ~30% in B-BYF. This increase in volume might be one of the reasons for the lower mechanical strengths of B-BYF mortars, jointly with the lower degree of hydration of main phases. The stratlingite/katoite ratio in both systems increases by increasing the w/c ratio. However, the impact of these results on the evolution of the microstructure of hydrating materials should be determined by quantitative characterisation techniques, which have to be developed. It cannot be stated that the formation of C-S-H or C-A-S-H gel is favoured in st-BYF or in B-BYF, since ^29^Si MAS-NMR spectra are very similar.

Finally, the higher degree of reaction of β-C_2_S in st-BYF and the optimisation of processing, in terms of superplasticiser content, have resulted key to achieving competitive compressive strengths. St-BYF mortars show compressive strength values >40 MPa at 7 days of hydration, and at 28 days, this value increases to >50 MPa for plain st-BYF and >70 MPa for st-BYF with B_2_O_3_ as admixture. These values are comparable (or higher) to mortars with type I 42.5R Portland cement.

## Figures and Tables

**Table 1:** Elemental compositions determined by XRF, expressed in weight percentage of oxides for both BYF clinkers, including loss on ignition (LOI).

Clinker	CaO	SiO_2_	SO_3_	Al_2_O_3_	Fe_2_O_3_	MgO	TiO_2_	K_2_O	Na_2_O	B_2_O_3_	LOI
st-BYF	51.8	17.0	3.5	18.6	6.4	1.0	0.6	0.3	-	-	0.8
B-BYF	51.7	15.1	5.3	16.6	6.0	1.0	0.5	0.3	0.9 *	2.0 *	0.6

* Nominally added as borax.

**Table 2:** RQPA, including ACn and free water (FW) of st-BYF with w/c = 0.40 and 0.3 wt % of SP (st-BYF-wc040_03SP), per 100 g of anhydrous cement. Density of all phases and stratlingite/katoite ratio are also included.

Phase	t_0_	7 Days	28 Days	120 Days	ρ (cm^3^/g)
β-C_2_S	28.1	19.8	12.3	11.1	3.29
γ-C_2_S	4.1	2.3	2.7	3.5	2.97
o-C_4_A_3_ $\bar{S}$	14.8	--	--	--	2.60
C_4_AF	13.5	1.2	1.3	1.5	3.73
C_2_AS	2.4	1.7	1.9	2.0	3.01
C$\bar{S}$	8.3	--	--	--	2.98
Ettringite	--	16.2	15.2	18.2	1.78
Katoite	--	9.5	10.4	13.0	2.70
Stratlingite	--	10.2	8.5	7.4	1.86
AFm	--	0.7	0.7	0.6	1.77
ACn	28.8	72.7	84.7	82.6	3.25 ^#^/2.00
FW	40.0	5.6	3.0	0.0	1.00
Strat/Kat	0.0	1.1	0.8	0.6	

^#^ Value used for the initial ACn of t_0_ column.

**Table 3:** RQPA, including ACn and FW of st-BYF with w/c = 0.40, 0.1 wt % of SP and 0.25 wt % of B_2_O_3_ added as borax (st-BYF-wc040_01SP_0.25B_2_O_3_), per 100 g of anhydrous cement. Stratlingite/katoite ratio is also included.

Phase	t_0_	7 Days	28 Days	120 Days
β-C_2_S	28.1	24.7	14.6	12.4
γ-C_2_S	4.1	2.9	2.5	2.9
o-C_4_A_3_ $\bar{S}$	14.8	--	--	--
C_4_AF	13.5	2.4	0.7	1.0
C_2_AS	2.4	1.8	1.9	2.3
C$\bar{S}$	8.3	1.6	1.0	1.2
Ettringite	--	16.3	19.4	18.3
Katoite	--	8.4	14.9	18.2
Stratlingite	--	8.1	10.1	9.0
ACn	28.8	69.0	74.8	74.7
FW	40.0	4.8	0.1	0.0
Strat/Kat	0.0	1.0	0.7	0.5

**Table 4:** RQPA, including ACn and FW of st-BYF with w/c = 0.50 (st-BYF-wc050), per 100 g of anhydrous cement. Stratlingite/katoite ratio is also included.

Phase	t_0_	7 Days	28 Days	120 Days
β-C_2_S	28.1	16.1	11.6	6.3
γ-C_2_S	4.1	3.5	3.9	4.0
o-C_4_A_3_ $\bar{S}$	14.8	--	--	--
C_4_AF	13.5	2.4	1.6	1.3
C_2_AS	2.4	2.0	2.2	1.9
C$\bar{S}$	8.3	--	--	--
Ettringite	0.0	13.1	12.3	16.7
Katoite	0.0	7.9	8.7	10.0
Stratlingite	0.0	9.1	8.7	6.2
AFm	0.0	1.2	2.3	2.5
ACn	28.8	82.0	86.8	95.5
FW	50.0	12.7	12.0	5.7
Strat/Kat	0.0	1.2	1.0	0.6

**Table 5:** RQPA, including ACn and FW of st-BYF with w/c = 0.60 (st-BYF-wc060), per 100 g of anhydrous cement. Stratlingite/katoite ratio is also included.

Phase	t_0_	7 Days	28 Days	120 Days
β-C_2_S	28.1	15.5	12.4	8.2
γ-C_2_S	4.1	2.9	3.0	3.1
o-C_4_A_3_ $\bar{S}$	14.8	--	--	--
C_4_AF	13.5	1.7	1.2	1.0
C_2_AS	2.4	2.0	1.6	1.7
C$\bar{S}$	8.3	--	--	--
Ettringite	--	6.7	17.4	16.4
Katoite	--	5.4	7.5	9.9
Stratlingite	--	10.0	9.6	6.8
AFm	--	1.8	3.5	4.4
ACn	28.8	89.2	83.4	93.1
FW	60.0	24.8	20.5	15.6
Strat/Kat	0.0	1.9	1.3	0.7

**Table 6:** RQPA, including ACn and FW of B-BYF with w/c = 0.40 and 0.4 wt % of SP (B-BYF-wc040_04SP), per 100 g of anhydrous cement. Density of all phases and stratlingite/katoite ratio are also included.

Phase	t_0_	7 Days	28 Days	120 Days	ρ (cm^3^/g)
α’_H_-C_2_S	29.4	23.2	23.7	14.7	3.21
γ-C_2_S	0.4	0.5	0.6	0.4	2.97
cub-C_4_A_3_ $\bar{S}$	17.0	1.7	1.7	1.1	2.60
C_4_AF	15.0	11.6	12.2	8.6	3.73
C_2_AS	0.7	--	--	--	3.01
C$\bar{S}$	8.0	--	--	--	2.98
Ettringite	--	22.8	25.9	30.8	1.78
Katoite	--	0.8	0.7	3.5	2.70
Stratlingite	--	--	--	--	1.86
ACn	29.5	67.3	65.7	81.0	3.25 ^#^/2.00
FW	40.0	12.1	9.5	0.0	1.00
Strat/Kat	0.0	0.0	0.0	0.0	

^#^ Value used for the initial ACn of t_0_ column.

**Table 7:** RQPA, including ACn and FW of B-BYF with w/c = 0.50 and 0.3 wt % of SP (B-BYF-wc050_03SP), per 100 g of anhydrous cement. Stratlingite/katoite ratio is also included.

Phase	t_0_	7 Days	28 Days	120 Days
α’_H_-C_2_S	29.4	25.1	21.6	13.3
γ-C_2_S	0.4	0.5	0.4	0.7
cub-C_4_A_3_ $\bar{S}$	17.0	1.5	0.9	0.5
C_4_AF	15.0	12.0	10.8	8.6
C_2_AS	0.7	--	--	--
C$\bar{S}$	8.0	--	--	--
Ettringite	--	21.8	24.9	29.9
Katoite	--	--	--	--
Stratlingite	--	0.0	2.5	3.6
CN$\bar{S}$ **	--	0.8	--	--
Hc ^$^	--	--	--	0.3
AFm	--	--	0.6	0.6
ACn	29.5	66.7	72.7	87.4
FW	50.0	21.7	15.6	5.2
Strat/Kat	--	--	--	--

** Ca_0.857_Na_0.285_(SO_4_)(H_2_O)_0.473_; ^$^ hemicarboaluminate.

**Table 8:** RQPA, including ACn and FW of B-BYF with w/c = 0.60 (B-BYF-wc060), per 100 g of anhydrous cement. Stratlingite/katoite ratio is also included.

Phase	t_0_	7 Days	28 Days	120 Days
α’_H_-C_2_S	29.4	25.6	21.3	17.2
γ-C_2_S	0.4	0.7	0.7	0.4
cub-C_4_A_3_ $\bar{S}$	17.0	0.5	--	--
C_4_AF	15.0	13.3	12.4	12.8
C_2_AS	0.7	--	--	--
C$\bar{S}$	8.0	--	--	--
Ettringite	--	21.6	27.3	33.4
Katoite	--	--	0.8	1.2
Stratlingite	--	0.5	4.2	5.0
CN$\bar{S}$ **	--	2.4	0.8	0.6
AFm	--	0.5	1.0	0.5
ACn	29.5	64.7	65.6	68.5
FW	60.0	30.2	25.8	20.4
Strat/Kat	0.0	--	5.1	4.3

** Ca_0.857_Na_0.285_(SO_4_)(H_2_O)_0.473_.

## Data Availability

All the LXRPD raw patterns analysed in this article are openly deposited in Zenodo at 10.5281/zenodo.4926032.

## Supplementary Materials

The following are available online at https://www.mdpi.com/article/10.3390/ma14174862/s1. Figure S1: PSD of st-BYF and B-BYF, Figures S2–S8: LXRPD raw patterns of hydrated pastes, Figures S9–S11: Rietveld plots of selected pastes hydrated at 7, 28 and 120 d.

## References

[1] Shi C., Qu B., Provis J.L. (2019). Recent progress in low-carbon binders. Cem. Concr. Res..

[2] Amato I. (2013). Green cement: Concrete solutions. Nature.

[3] Gartner E.M. What are BYF cements, and how do they differ from CSA cements?. Proceedings of the The Future of Cement, 200 years after Louis Vicat.

[4] Aranda M.A.G., De la Torre A.G., Pacheco-Torgal F., Jalali S., Labrincha J. (2013). Sulfoaluminate cement. Eco-Efficient Concrete.

[5] Zajac M., Skocek J., Stabler C., Bullerjahn F., Ben Haha M. (2019). Hydration and performance evolution of belite-ye’elimite-ferrite cement. Adv. Cem. Res..

[6] Quillin K. (2001). Performance of belite-sulfoaluminate cements. Cem. Concr. Res..

[7] Julphunthong P., Joyklad P. (2019). Utilization of several industrial wastes as raw material for calcium sulfoaluminate cement. Materials.

[8] Su D., Yue G., Li Q., Guo Y., Gao S., Wang L. (2019). Research on the preparation and properties of high belite sulphoaluminate cement (HBSAC) based on various industrial solid wastes. Materials.

[9] Cuesta A., Ayuela A., Aranda M.A.G. (2021). Belite cements and their activation. Cem. Concr. Res..

[10] Frigione G., Zenone F., Esposito M.V. (1983). The effect of chemical composition on portland cement clinker grindability. Cem. Concr. Res..

[11] Morsli K., De la Torre A.G., Zahir M., Aranda M.A.G. (2007). Mineralogical phase analysis of alkali and sulfate bearing belite rich laboratory clinkers. Cem. Concr. Res..

[12] Staněk T., Sulovský P. (2015). Active low-energy belite cement. Cem. Concr. Res..

[13] Morin V., Walenta G., Gartner E., Termkhajornkit P., Baco I., Casabonne J.M. Hydration of a Belite-Calcium Sulfoaluminate-Ferrite cement: Aether^TM^. Proceedings of the 13th International Congress on the Chemistry of Cement.

[14] Álvarez-Pinazo G., Santacruz I., León-Reina L., Aranda M.A.G., De La Torre A.G. (2013). Hydration reactions and mechanical strength developments of iron-rich sulfobelite eco-cements. Ind. Eng. Chem. Res..

[15] Bullerjahn F., Zajac M., Skocek J., Ben Haha M. (2019). The role of boron during the early hydration of belite ye’elimite ferrite cements. Constr. Build. Mater..

[16] Kacimi L., Simon-Masseron A., Salem S., Ghomari A., Derriche Z. (2009). Synthesis of belite cement clinker of high hydraulic reactivity. Cem. Concr. Res..

[17] Chatterjee A.K. (1996). High belite cements—Present status and future technological options: Part I. Cem. Concr. Res..

[18] Morin V., Termkhajornkit P., Huet B., Pham G. (2017). Impact of quantity of anhydrite, water to binder ratio, fineness on kinetics and phase assemblage of belite-ye’elimite-ferrite cement. Cem. Concr. Res..

[19] Bullerjahn F., Zajac M., Ben Haha M., Scrivener K.L. (2019). Factors influencing the hydration kinetics of ye’elimite; effect of mayenite. Cem. Concr. Res..

[20] Andrade Neto J.D.S., De la Torre A.G., Kirchheim A.P. (2021). Effects of sulfates on the hydration of Portland cement—A review. Constr. Build. Mater..

[21] Su D., Li Q., Guo Y., Yue G., Wang L. (2020). Effect of residual CaSo4 in clinker on properties of high belite sulfoaluminate cement based on solid wastes. Materials.

[22] Álvarez-Pinazo G., Santacruz I., Aranda M.A.G., De la Torre A.G. (2016). Hydration of belite–ye’elimite–ferrite cements with different calcium sulfate sources. Adv. Cem. Res..

[23] Dunster A., Quillin K., Tipple C., Albert B., Comparet C., Gartner E., Walenta G. (2016). Performance and Durability of Concrete Made using Lower Carbon Belite-ye’ Elimite-Ferrite Cement (BR 512).

[24] Zhang J., Ke G., Liu Y. (2021). Early hydration heat of calcium sulfoaluminate cement with influences of supplementary cementitious materials and water to binder ratio. Materials.

[25] Koga G.Y., Albert B., Nogueira R.P. (2020). On the hydration of Belite-Ye’elimite-Ferrite (BYF) cement pastes: Effect of the water-to-cement ratio and presence of fly ash. Cem. Concr. Res..

[26] John V.M., Quattrone M., Abrão P.C.R.A., Cardoso F.A. (2019). Rethinking cement standards: Opportunities for a better future. Cem. Concr. Res..

[27] Termkhajornkit P., Vu Q.H., Barbarulo R., Daronnat S., Chanvillard G. (2014). Dependence of compressive strength on phase assemblage in cement pastes: Beyond gel–space ratio—Experimental evidence and micromechanical modeling. Cem. Concr. Res..

[28] Sha S., Wang M., Shi C., Xiao Y. (2020). Influence of the structures of polycarboxylate superplasticizer on its performance in cement-based materials-A review. Constr. Build. Mater..

[29] Zhu W., Feng Q., Luo Q., Bai X., Lin X., Zhang Z. (2021). Effects of PCE on the Dispersion of Cement Particles and Initial Hydration. Materials.

[30] Link J., Sowoidnich T., Pfitzner C., Gil-Diaz T., Heberling F., Lützenkirchen J., Schäfer T., Ludwig H.M., Haist M. (2020). The influences of cement hydration and temperature on the thixotropy of cement paste. Materials.

[31] Álvarez-Pinazo G., Cuesta A., García-Maté M., Santacruz I., Losilla E.R., De la Torre A.G., León-Reina L., Aranda M.A.G. (2012). Rietveld quantitative phase analysis of Yeelimite-containing cements. Cem. Concr. Res..

[32] Von Dreele R.B., Larson A.C. (2004). General structure analysis system (GSAS). Los Alamos Natl. Lab. Rep. LAUR.

[33] Thompson P., Cox D.E., Hastings J.B. (1987). Rietveld Refinement of Debye-Scherrer Synchrotron X-ray Data from A1203. J. Appl. Crystallogr..

[34] Finger L.W., Cox D.E., Jephcoat A.P. (1994). Correction for powder diffraction peak asymmetry due to axial divergence. J. Appl. Crystallogr..

[35] De la Torre A.G., Santacruz I., Cuesta A., León-Reina L., Aranda M.A.G., Pöllmann H. (2017). Diffraction and crystallography applied to anhydrous cements. Cementitious Materials.

[36] Aranda M.A.G., Cuesta A., De la Torre A.G., Santacruz I., León-Reina L., Pöllmann H. (2017). Diffraction and crystallography applied to hydrating cements. Cementitious Materials.

[37] De la Torre A.G., Bruque S., Aranda M.A.G. (2001). Rietveld quantitative amorphous content analysis. J. Appl. Crystallogr..

[38] Zea-Garcia J.D., De la Torre A.G., Aranda M.A.G., Santacruz I. (2020). Processing optimisation and characterisation of standard and doped alite-belite-ye’elimite ecocement pastes and mortars. Cem. Concr. Res..

[39] Wadsö L. (2010). Operational issues in isothermal calorimetry. Cem. Concr. Res..

[40] García-Maté M., Londono-Zuluaga D., De la Torre A.G., Losilla E.R., Cabeza A., Aranda M.A.G., Santacruz I. (2016). Tailored setting times with high compressive strengths in bassanite calcium sulfoaluminate eco-cements. Cem. Concr. Compos..

[41] Emoto T., Bier T.A. (2007). Rheological behavior as influenced by plasticizers and hydration kinetics. Cem. Concr. Res..

[42] Winnefeld F., Lothenbach B. (2010). Hydration of calcium sulfoaluminate cements—Experimental findings and thermodynamic modelling. Cem. Concr. Res..

[43] Dilnesa B.Z., Wieland E., Lothenbach B., Dähn R., Scrivener K.L. (2014). Fe-containing phases in hydrated cements. Cem. Concr. Res..

[44] Cuesta A., De la Torre A.G., Santacruz I., Diaz A., Trtik P., Holler M., Lothenbach B., Aranda M.A.G. (2019). Quantitative disentanglement of nanocrystalline phases in cement pastes by synchrotron ptychographic X-ray tomography. IUCrJ.

[45] Álvarez-Pinazo G., Cuesta A., García-Maté M., Santacruz I., Losilla E.R., Sanfélix S.G., Fauth F., Aranda M.A.G., De La Torre A.G. (2014). In-situ early-age hydration study of sulfobelite cements by synchrotron powder diffraction. Cem. Concr. Res..

[46] Wadsö L., Winnefeld F., Riding K., Sandberg P., Scrivener K.L., Snellings R., Lothenbach B. (2016). Calorimetry. A Practical Guide to Microstructural Analysis of Cementitious Materials.

[47] Sanfélix S.G., Zea-Garcia J.D., Londono-Zuluaga D., Santacruz I., De la Torre A.G., Kjøniksen A.-L. (2020). Hydration development and thermal performance of calcium sulphoaluminate cements containing microencapsulated phase change materials. Cem. Concr. Res..

[48] Zhang G., Li G., Li Y. (2016). Effects of superplasticizers and retarders on the fluidity and strength of sulphoaluminate cement. Constr. Build. Mater..

[49] Cuesta A., De la Torre A.G., Santacruz I., Trtik P., da Silva J.C., Diaz A., Holler M., Aranda M.A.G. (2017). Chemistry and Mass Density of Aluminum Hydroxide Gel in Eco-Cements by Ptychographic X-ray Computed Tomography. J. Phys. Chem. C.

[50] Paul G., Boccaleri E., Cassino C., Gastaldi D., Buzzi L., Canonico F., Marchese L. (2021). Fingerprinting the Hydration Products of Hydraulic Binders Using Snapshots from Time-Resolved In Situ Multinuclear MAS NMR Spectroscopy. J. Phys. Chem. C.

[51] Mohamed A.K., Moutzouri P., Berruyer P., Walder B.J., Siramanont J., Harris M., Negroni M., Galmarini S.C., Parker S.C., Scrivener K.L. (2020). The Atomic-Level Structure of Cementitious Calcium Aluminate Silicate Hydrate. J. Am. Chem. Soc..

[52] Cuesta A., Ichikawa R., Londono-Zuluaga D., De la Torre A.G., Santacruz I., Turrillas X., Aranda M.A.G. (2017). Aluminum hydroxide gel characterization within a calcium aluminate cement paste by combined Pair Distribution Function and Rietveld analyses. Cem. Concr. Res..

[53] Londono-Zuluaga D., Tobón J.I., Aranda M.A.G., Santacruz I., De la Torre A.G. (2018). Influence of fly ash blending on hydration and physical behavior of belite–alite–ye’elimite cements. Mater. Struct..

[54] Morsli K., De la Torre A.G., Stöber S., Cuberos A.J.M., Zahir M., Aranda M.A.G. (2007). Quantitative phase analysis of laboratory-active belite clinkers by synchrotron powder diffraction. J. Am. Ceram. Soc..

[55] Shirani S., Cuesta A., Morales-Cantero A., De la Torre A.G., Olbinado M.P., Aranda M.A.G. (2021). Influence of curing temperature on belite cement hydration: A comparative study with Portland cement. Cem. Concr. Res..

[56] Mumme W.G., Hill R.J., Bushnell-Wye G., Segnit E.R. (1995). Rietveld crystal structure refinements, crystal chemistry and calculated powder diffraction data for the polymorphs of dicalcium silicate and related phases. Neues Jahrb. Fuer Mineral..

[57] Walenta G., Comparet C., Morin V., Gartner E. (2019). Hydraulic binder based on sulfoaluminate clinker and minerals additions. European Patent.

[58] Gartner E., Li G. (2010). High Belite-Containing Sulfoaluminous Clinker, Method for the Production and the Use Thereof for Preparing Hydraulic Binders. U.S. Patent.

[59] Dai Z., Tran T.T., Skibsted J. (2014). Aluminum Incorporation in the C-S-H Phase of White Portland Cement-Metakaolin Blends Studied by ^27^ Al and ^29^ Si MAS NMR Spectroscopy. J. Am. Ceram. Soc..

[60] Santacruz I., De la Torre A.G., Álvarez-Pinazo G., Cabeza A., Cuesta A., Sanz J., Aranda M.A.G. (2016). Structure of stratlingite and effect of hydration methodology on microstructure. Adv. Cem. Res..

[61] Pedersen M.T. (2018). Characterization of Ye’elimite and Calcium Sulfo-Aluminate Cement Incorporating Calcined Clays. Ph.D. Thesis.

[62] Andersen M.D., Jakobsen H.J., Skibsted J. (2003). Incorporation of aluminum in the calcium silicate hydrate (C-S-H) of hydrated Portland cements: A high-field 27Al and 29Si MAS NMR investigation. Inorg. Chem..

[63] Chandler H.W., MacPhee D.E., Atkinson I., Henderson R.J., Merchant I.J. (2000). Enhancing the mechanical behaviour of cement based materials. J. Eur. Ceram. Soc..

[64] Chen I.A., Hargis C.W., Juenger M.C.G. (2012). Understanding expansion in calcium sulfoaluminate-belite cements. Cem. Concr. Res..

[65] Bernal I.M.R., Shirani S., Cuesta A., Santacruz I., Aranda M.A.G. (2021). Phase and microstructure evolutions in LC 3 binders by multi-technique approach including synchrotron microtomography. Constr. Build. Mater..

[66] Cao S., Yilmaz E., Yin Z., Xue G., Song W., Sun L. (2021). CT scanning of internal crack mechanism and strength behavior of cement-fiber-tailings matrix composites. Cem. Concr. Compos..

