# Supplementary material for: Effect of Boron and Water-to-Cement Ratio on the Performances of Laboratory Prepared Belite-Ye’elimite-Ferrite (BYF) Cements
Source: Materials (Basel). 2021 Aug 26;14(17):4862. doi: 10.3390/ma14174862 (PMC8432724; doi:10.3390/ma14174862)
Supplement: Supplementary file 1 [file materials-14-04862-s001.zip › materials-1336349-supplementary.pdf]

materials

MDPI

Supporting information

Effect of boron and water-to-cement ratio on the performances of laboratory prepared Belite-Ye'elimite-Ferrite (BYF) cements.

Raquel Pérez-Bravo[1], Alejandro Morales-Cantero[1], Margherita Bruscolini[2], Miguel A.G. Aranda[1], Isabel Santacruz[1], Angeles G. De la Torre[1,a]

This supporting information contains eleven figures: PSD of st-BYF and B-BYF (Figure S1), LXRPD raw patterns of hydrated pastes (Figures S2 to S8) and Rietveld plots of selected pastes hydrated at 7, 28 and 120 d (Figures S9 to S11).

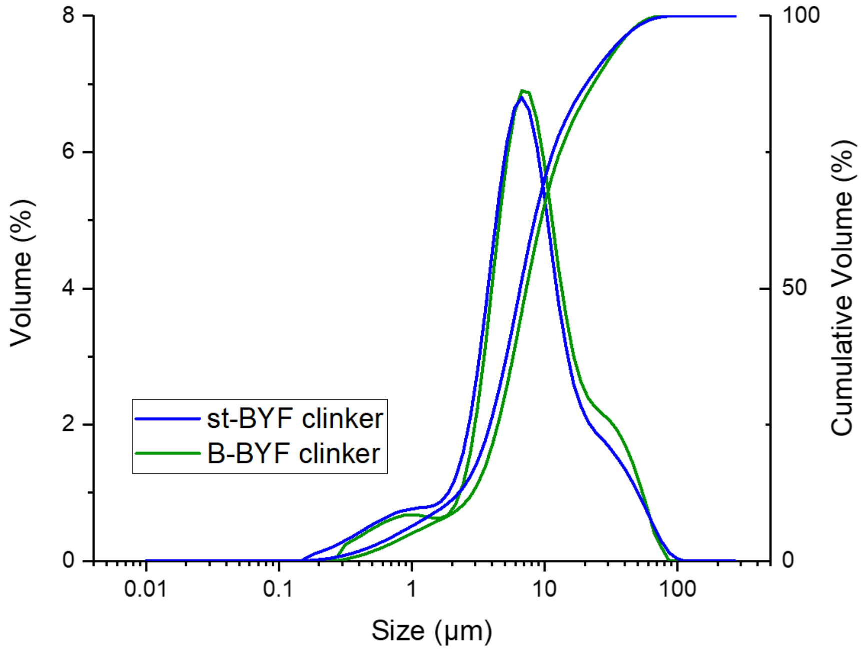

Figure S1. Particle size distribution of BYF clinkers.

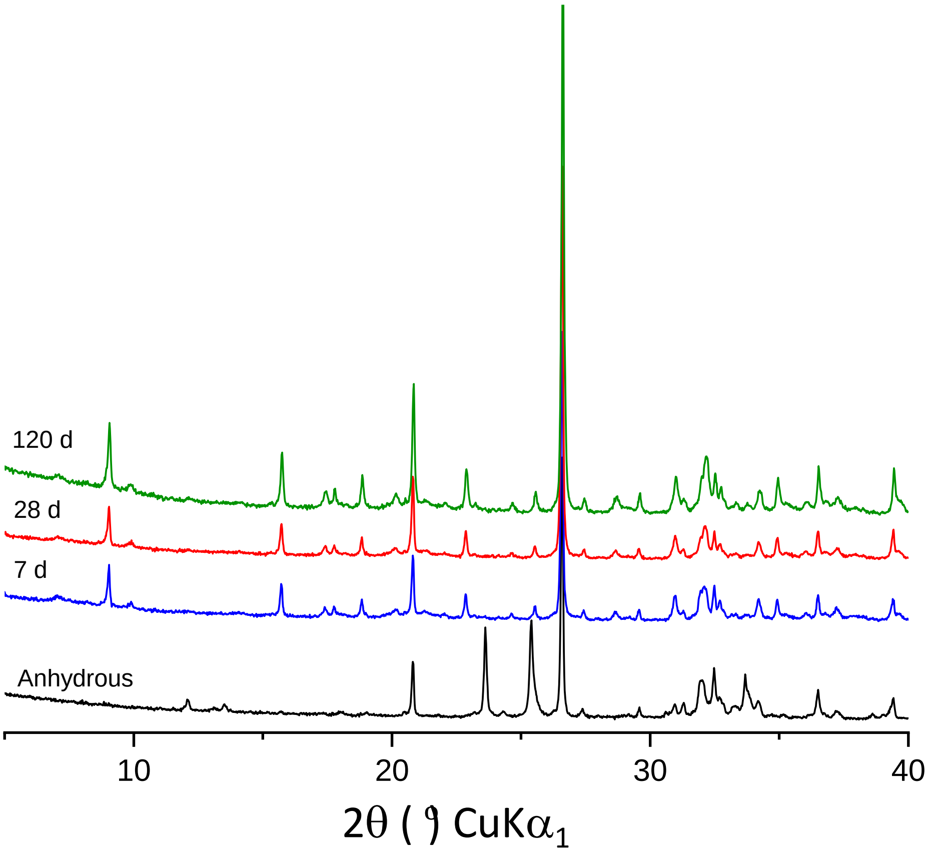

Figure S2. Raw LXRPD data of st-BYF_wc040_03SP.

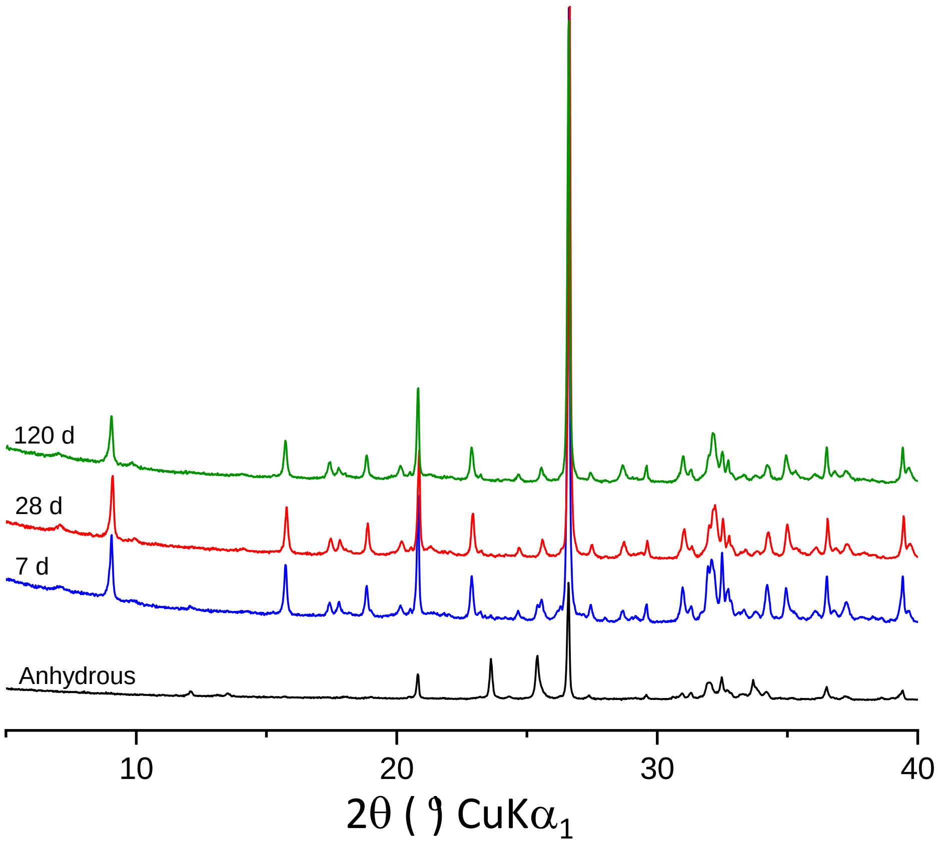

Figure S3. Raw LXRPD data of st-BYF_wc040_01SP_0.25B_2O_3.

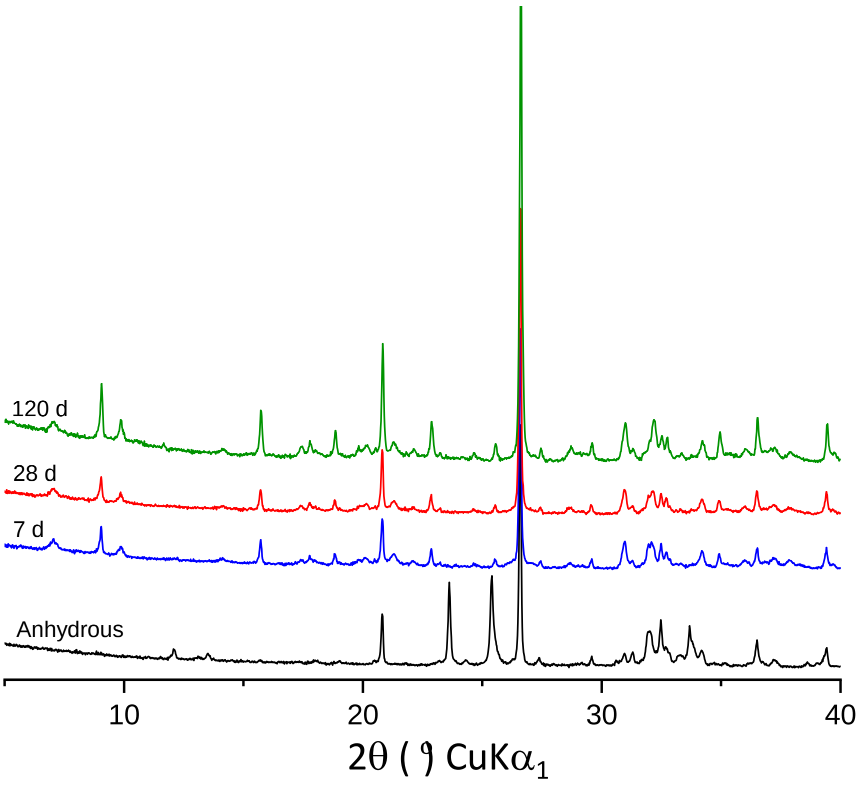

Figure S4. Raw LXRPD data of st-BYF_wc050.

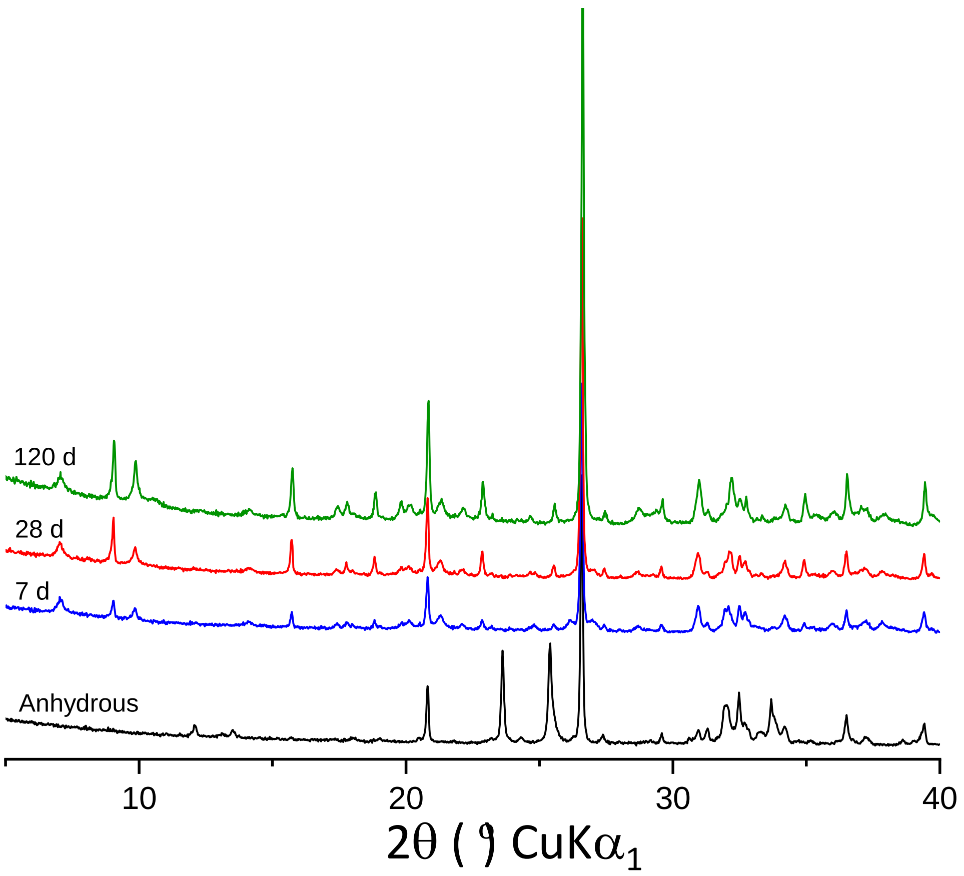

Figure S5. Raw LXRPD data of st-BYF_wc060.

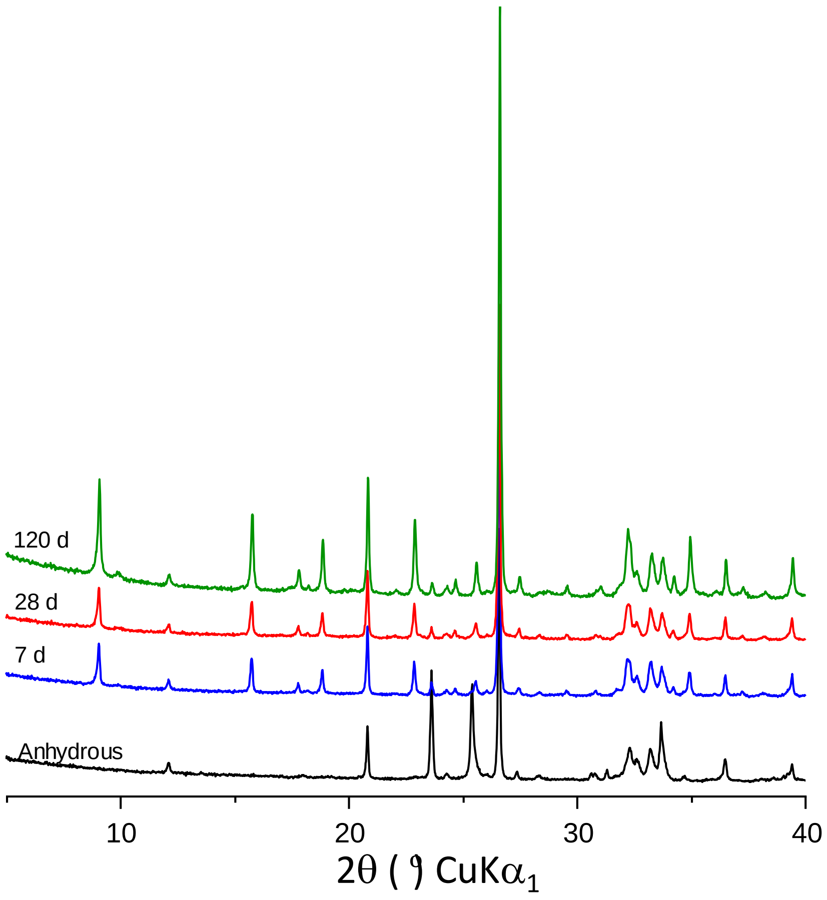

Figure S6. Raw LXRPD data of B-BYF_wc040_04SP.

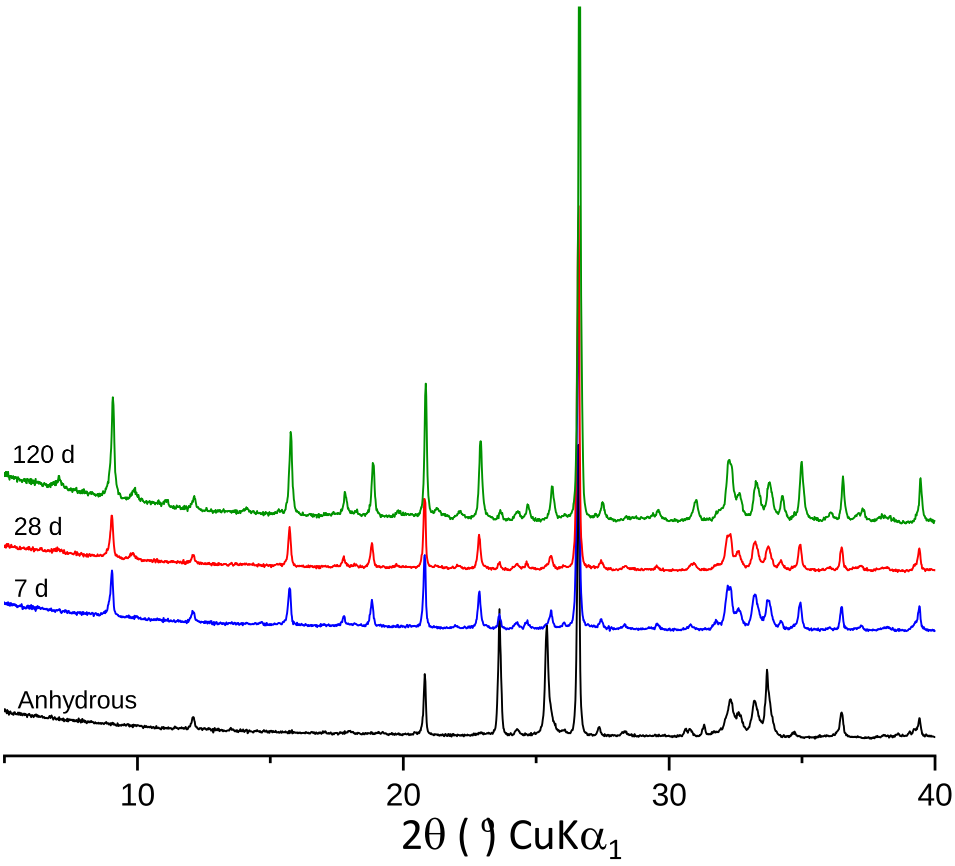

Figure S7. Raw LXRPD data of B-BYF_wc050_03SP.

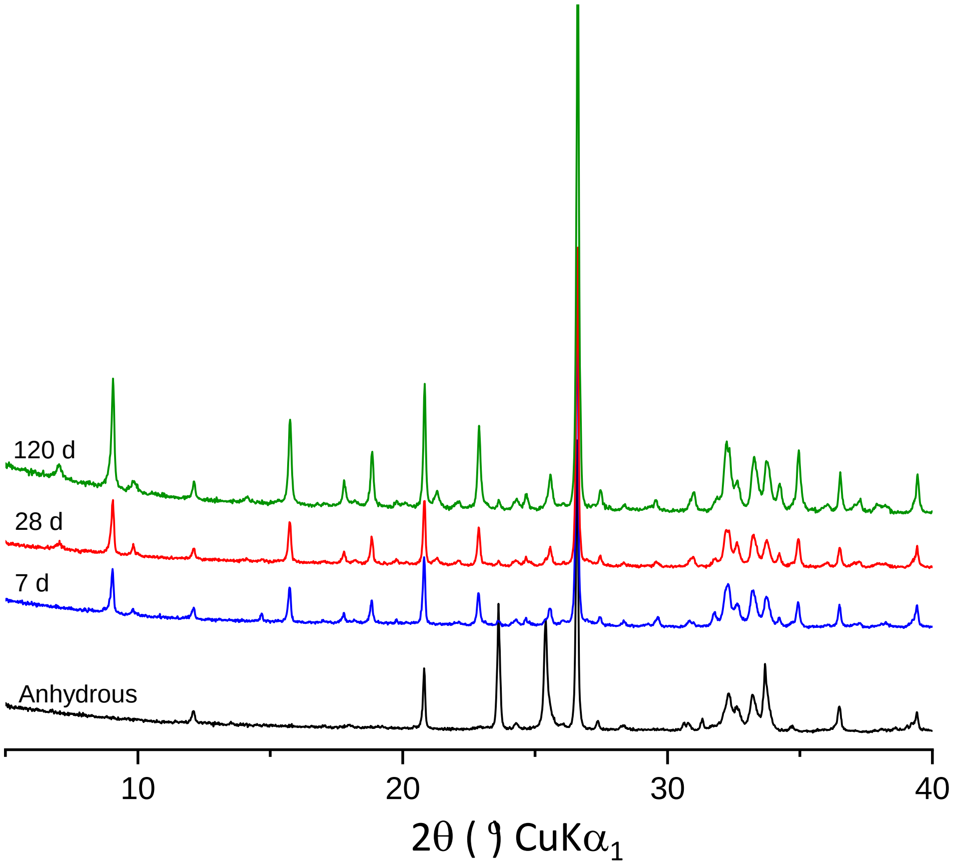

Figure S8. Raw LXRPD data of B-BYF_wc060.

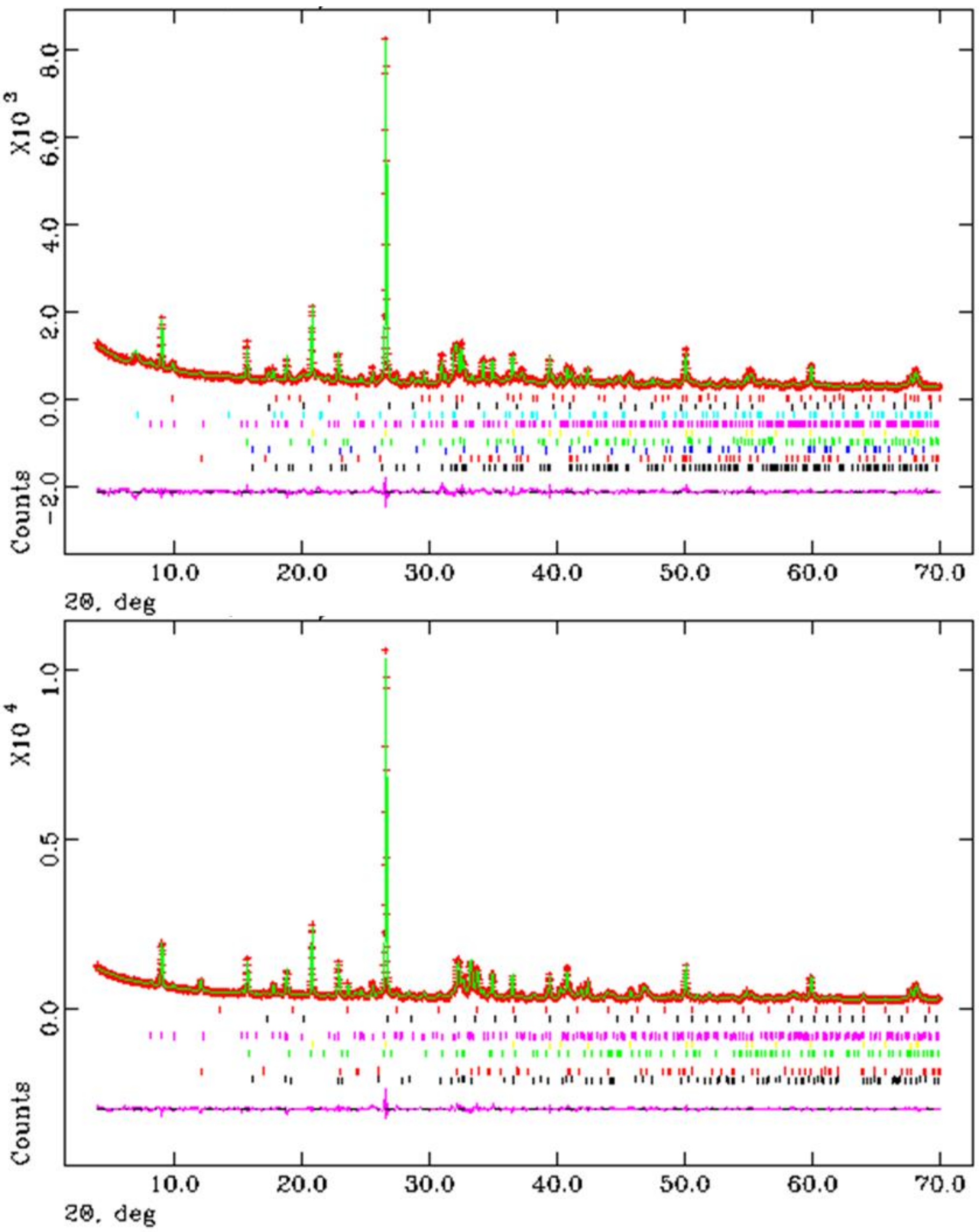

Figure S9. Rietveld plots of st-BYF_wc040_03SP (top) and B-BYF_wc040_04SP (bottom) at 7 days.

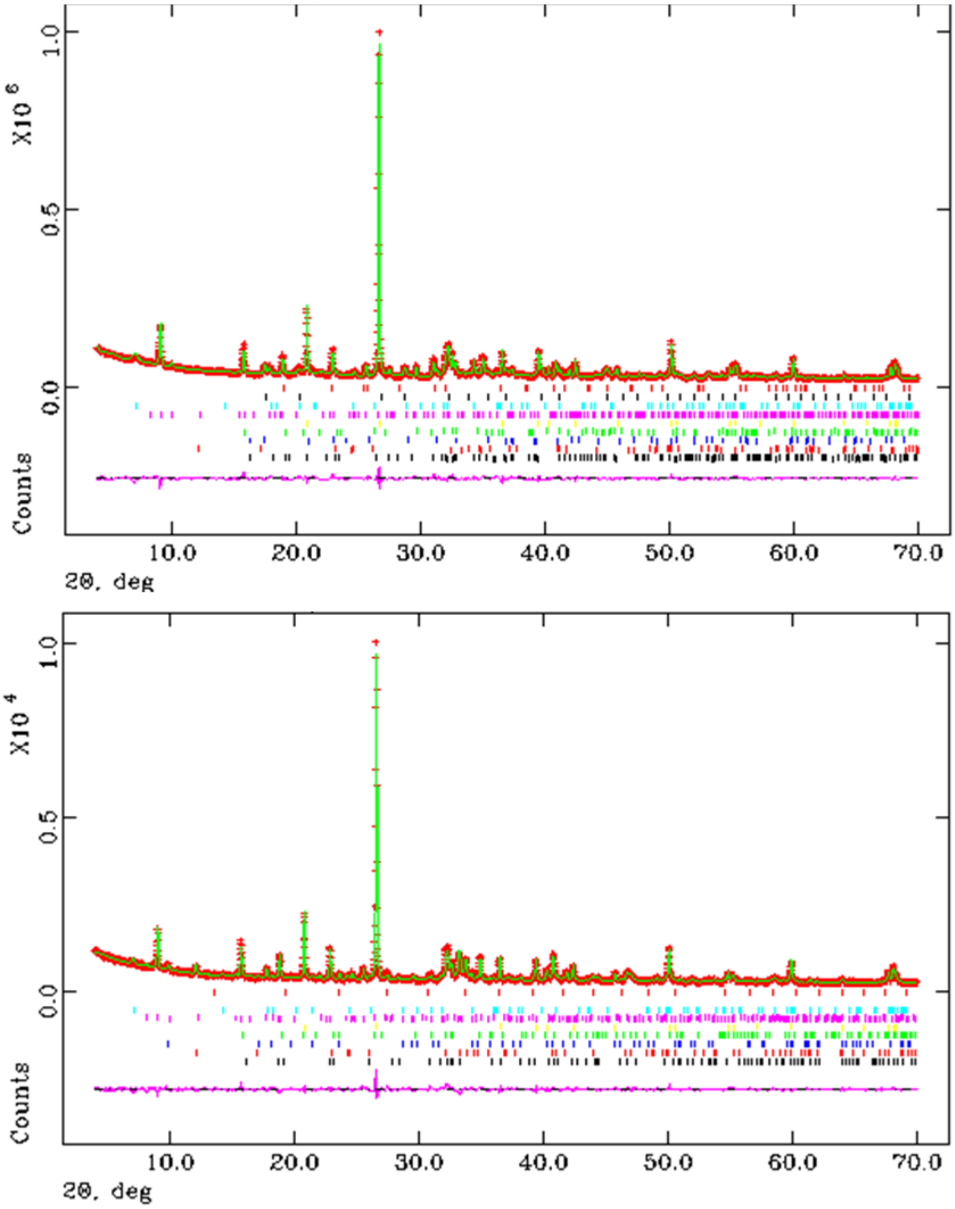

Figure S10. Rietveld plots of st-BYF_wc040_01SP_0.25B_2O_3 (top) and B-BYF_wc050_03SP (bottom) at 28 days.

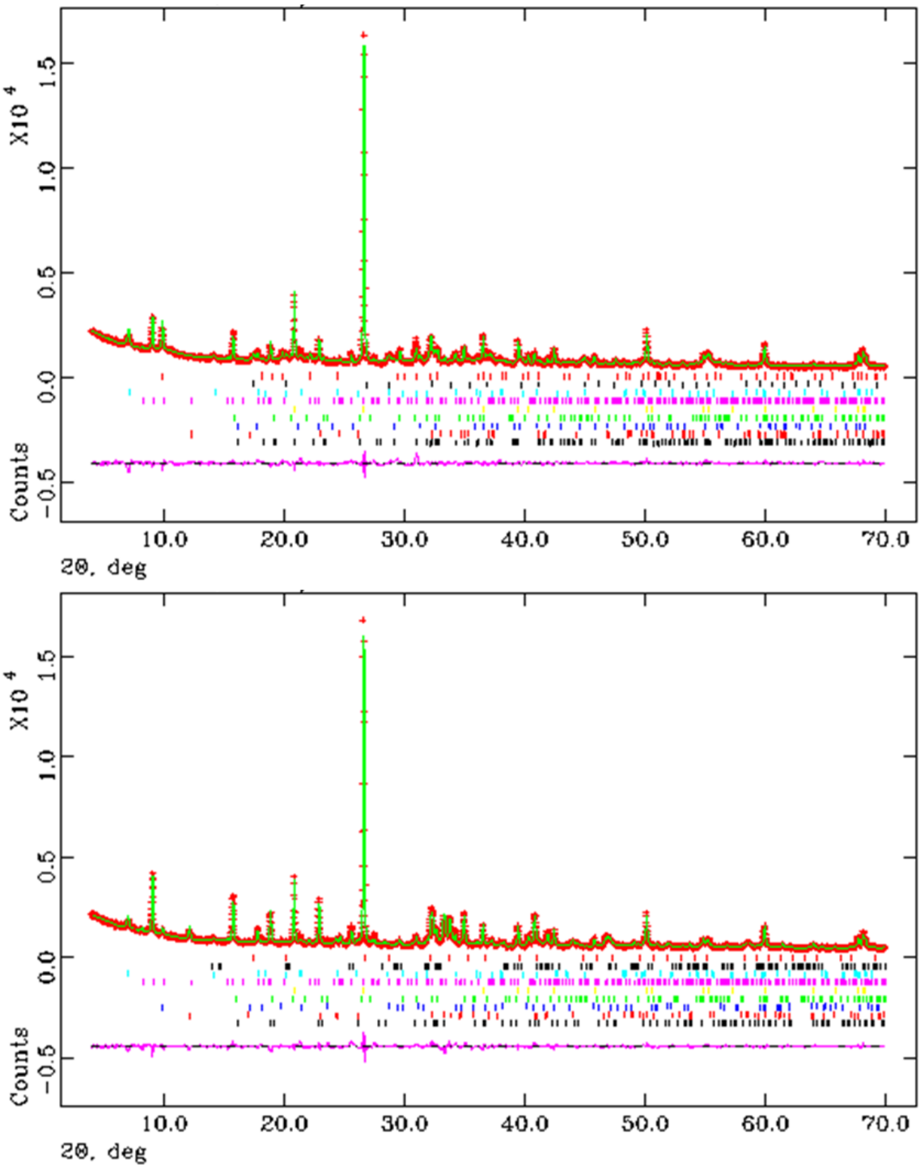

Figure S11. Rietveld plots of st-BYF_wc060 (top) and B-BYF_wc060 (bottom) at 120 days.
